# Overexpression of the Stress-Inducible *SsMAX2* Promotes Drought and Salt Resistance via the Regulation of Redox Homeostasis in *Arabidopsis*

**DOI:** 10.3390/ijms20040837

**Published:** 2019-02-15

**Authors:** Qiaojian Wang, Jun Ni, Faheem Shah, Wenbo Liu, Dongdong Wang, Yuanyuan Yao, Hao Hu, Shengwei Huang, Jinyan Hou, Songling Fu, Lifang Wu

**Affiliations:** 1 College of Forestry and Landscape Architecture, Anhui Agricultural University, Hefei 230000, Anhui, China; wangqj521@126.com (Q.W.); 15755059531@163.com (D.W.); 2 Key Laboratory of High Magnetic Field and Ion Beam Physical Biology, Hefei Institutes of Physical Science, Chinese Academy of Sciences, Hefei 230000, Anhui, China; faheemhorticulturist@gmail.com (F.S.); liuwenbo9261@sina.com (W.L.); 17355356851@163.com (Y.Y.); huhaoasd@mail.ustc.edu.cn (H.H.); swhuang@ipp.ac.cn (S.H.); jyhou@ipp.ac.cn (J.H.)

**Keywords:** *SsMAX2*, *Sapium sebiferum*, drought, osmotic stress, salt stress, redox homeostasis, strigolactones, ABA

## Abstract

Recent studies have demonstrated that strigolactones (SLs) also participate in the regulation of stress adaptation; however, the regulatory mechanism remains elusive. In this study, the homolog of *More Axillary Branches 2*, which encodes a key component in SL signaling, in the perennial oil plant *Sapium sebiferum* was identified and functionally characterized in *Arabidopsis*. The results showed that the expression of *SsMAX2* in *S. sebiferum* seedlings was stress-responsive, and *SsMAX2* overexpression (OE) in *Arabidopsis* significantly promoted resistance to drought, osmotic, and salt stresses. Moreover, *SsMAX2* OE lines exhibited decreased chlorophyll degradation, increased soluble sugar and proline accumulation, and lower water loss ratio in response to the stresses. Importantly, anthocyanin biosynthesis and the activities of several antioxidant enzymes, such as superoxide dismutase (SOD), peroxidase (POD), and ascorbate peroxidase (APX), were enhanced in the *SsMAX2* OE lines, which further led to a significant reduction in hydrogen peroxide levels. Additionally, the *SsMAX2* OE lines exhibited higher expression level of several abscisic acid (ABA) biosynthesis genes, suggesting potential interactions between SL and ABA in the regulation of stress adaptation. Overall, we provide physiological and biochemical evidence demonstrating the pivotal role of *SsMAX2* in the regulation of osmotic, drought, and salt stress resistance and show that *MAX2* can be a genetic target to improve stress tolerance.

## 1. Introduction

Abiotic stresses, such as drought, salt, cold, and flooding, significantly affect vegetative and reproductive growth and cause devastating yield losses each year. Plants have developed different coping mechanisms to deal with these stresses, mainly through the regulation of phytohormonal networks and dynamic changes of intracellular chemicals [1,2]. Phytohormones play a central role in the regulation of both vegetative and reproductive growth as well as adaptation to adverse growth conditions [3]. Hormones, such as abscisic acid (ABA), cytokinin, auxin, and salicylic acid (SA), have been proposed to be directly involved in the regulation of stress tolerance [4,5,6,7]. Strigolactones (SLs), which are a group of terpenoid compounds, play a key role in the regulation of shoot branching and the symbiosis with fungi in interactions with other hormones [8,9,10,11]. Recently, it was revealed that SLs also regulate plant adaptations to abiotic stresses [12,13]. In *Arabidopsis*, exogenous application of GR24, a SL analog, significantly improved salt and drought resistance, while the mutation of SL signaling gene *MAX2* made it sensitive to abiotic stresses [13,14]. However, the regulatory mechanism of SLs in stress tolerance still remains largely elusive.

In the perennial woody plant, the biological and molecular functions of strigolactones in the regulation of plant growth and stress adaptation have barely been studied. In the bioenergy plant *Jatropha curcas*, SLs antagonistically regulate the axillary bud outgrowth in interactions with cytokinin and gibberellin [15,16]. Several recent reports have also demonstrated that manipulation of the expression of SL biosynthesis genes can lead to significant change of the shoot branching phenotype in *Populus* and *Malus* [17,18], indicating that SLs have significant functions on the morphogenesis of woody plants. Plant growth and seed yield of woody plants are also threatened by abiotic stresses, such as salt, drought, and cold. Based on recent discoveries on the role of SLs in stress regulation in *Arabidopsis*, a functional study of the key genes in SL biosynthesis or signaling in woody plants would provide potential targets for genetic modifications to generate new cultivars with higher tolerance to abiotic stresses.

*Sapium sebiferum*, the seeds of which contain high level of fatty acids, has been considered as one of the most promising bioenergy plants. The oil from its seed coat and kernel can be manufactured into resources for lubricants, candles, cosmetics, and biodiesels [19,20]. It is widely distributed in most areas of China and even in the marginal land. However, while the plant adapts well to flooding and cold conditions, it is more sensitive to drought and salt stresses. The selection of high-yield cultivars with high resistance to drought and salt stresses is therefore the foremost goal in the molecular breeding of *Sapium sebiferum*. In this study, we identified that the expression of the SL signaling component *MAX2* was strongly responsive to abiotic stresses in *S. sebiferum* seedlings. Then, the biological functions of *SsMAX2* in the regulation of drought, osmotic, and salt tolerance were evaluated in *Arabidopsis*. The regulatory mechanism of *SsMAX2* to stress tolerance was further investigated at the physiological, molecular, and biochemical levels. This study not only reveals a pivotal role of *SsMAX2* in the regulation of drought, osmotic, and salt stress resistance but also provides evidence that *SsMAX2* can be a useful target for genetic engineering to produce stress-resistant plants.

## 2. Results

### 2.1. Gene Cloning of SsMAX2 from Sapium sebiferum Seedlings and the Gene Expression Profile in Response to Abiotic Stresses

**Figure 1:**
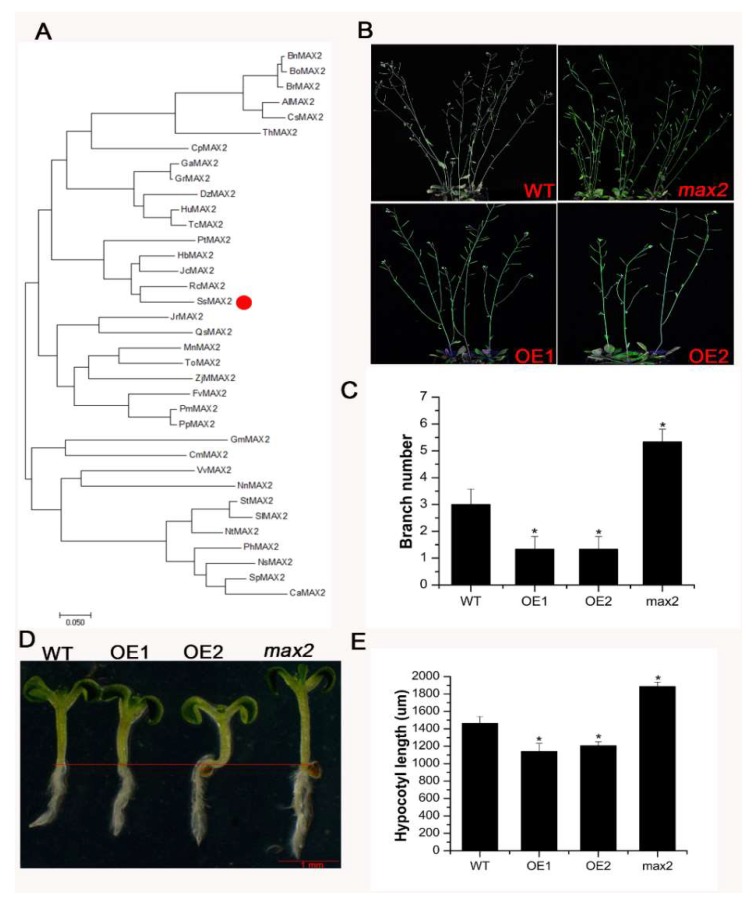
Phylogenetic analysis and functional characterization of *SsMAX2* in *Arabidopsis*. (**A**) Phylogenetic analysis of *SsMAX2* with its homologs from other species; (**B**,**C**) Rosette branching of 30-day-old plants of wild-type (WT), *SsMAX2* overexpression1 (OE1), OE2, and *max2*; (**D**,**E**) Hypocotyl length of 5-day-old seedlings of WT, *SsMAX2* OE1, OE2, and *max2* in half Murashige and Skoog (MS) medium. Data are presented as means ± SD of 20 replicates. Significant differences were determined by Student’s *t*-test. Significance level: * *p* < 0.05.

The MAX2 homolog with 76% sequence similarity with AtMAX2 was identified from the *S. sebiferum* transcriptome database (Figure 1A). Then, the phylogenetic analysis of the MAX2 sequences from more than 20 plant species was carried out (Table S1). The results showed that SsMAX2 had the highest sequence identity with several perennial woody plants, such as *Jatropha curcas*, *Ricinus communis*, and Hevea brasiliensis, which also belong to the Euphorbiaceae family (Figure 1A).

*MAX2* was the key component for SL signaling. Mutation of *MAX2* led to a significant increase in axillary branching and decrease in hypocotyl elongation. Our results are in accord with previous findings that constitutive expression of *SsMAX2* in *Arabidopsis* inhibits shoot branching and hypocotyl elongation, while *max2 Arabidopsis* mutant exhibits elongated hypocotyl growth (Figure 1B,C) and increased shoot branching (Figure 1D,E), demonstrating that *SsMAX2* has conserved functions with its homologs from *Arabidopsis*, rice, and pea.

**Figure 2:**
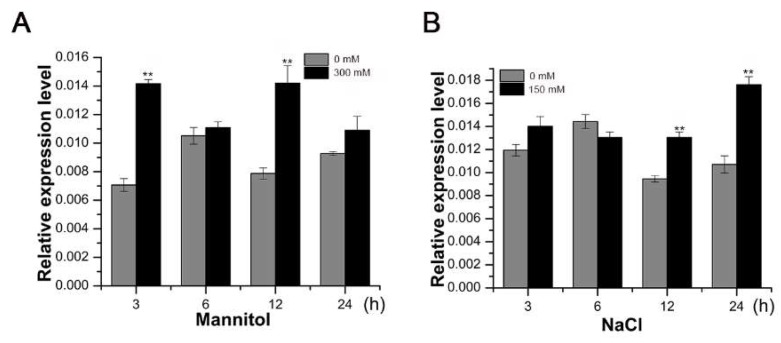
Expression profile of *SsMAX2* of 4-week-old *S. sebiferum* seedlings in response to (**A**) osmotic and (**B**) salt stresses. 300 mM mannitol and 150 mM NaCl were applied to 20-day-old *S. sebiferum* seedlings. *SsACT* was used as the internal control. Data are presented as means ± SD of three biological replicates. Significant differences were determined by Student’s *t*-test. Significance level: ** *p* < 0.01.

To investigate whether *SsMAX2* is involved in the regulation of abiotic regulation, we first characterized the time-course expression profile of *SsMAX2* of *S. sebiferum* seedlings in response to osmotic and salt stresses. The results showed that osmotic treatment, which was mimicked by mannitol, significantly increased *SsMAX2* expression at 3 h after treatment (Figure 2A), whereas salt treatment induced a significant increase in *SsMAX2* expression at 12 h (Figure 2B). This demonstrated that *SsMAX2* is a stress-responsive gene, which might be involved in the regulation of adaptation to abiotic stresses.

### 2.2. SsMAX2 Conferred Drought and Osmotic Stress Tolerance in Arabidopsis

**Figure 3:**
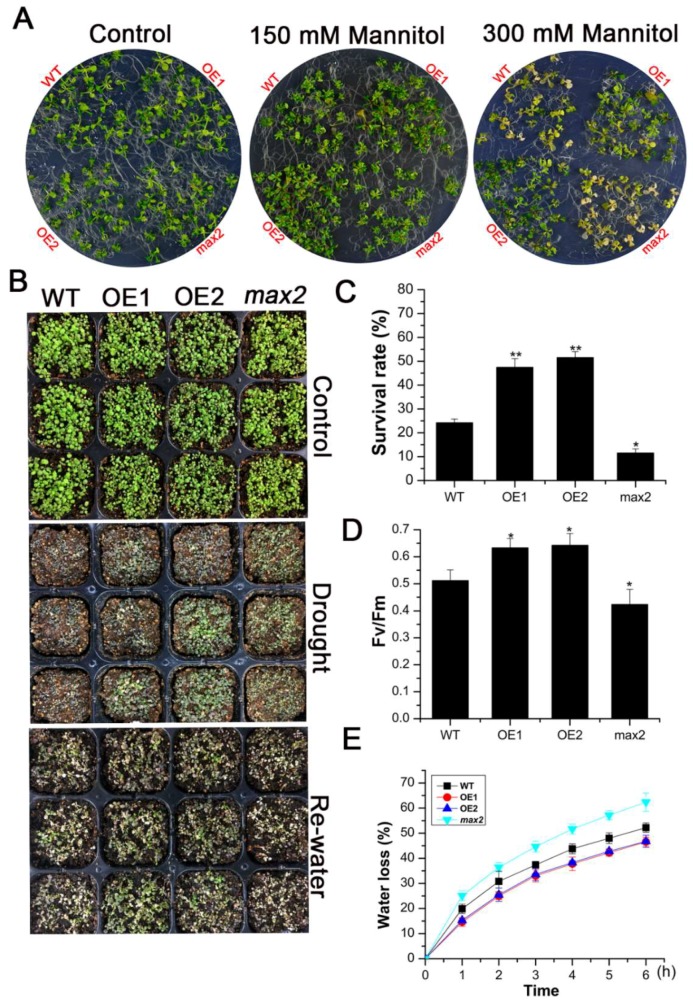
Constitutive expression of *SsMAX2* promoted osmotic and drought stress resistance in *Arabidopsis*. (**A**) Phenotype of *SsMAX2* OE lines, *max2*, and wild-type (WT) in half MS medium containing 150 and 300 mM mannitol; (**B**) Phenotype of different *Arabidopsis* lines after withholding water and rewatering treatment; (**C**) Survival rate of seedlings after drought treatment; (**D**) Maximum photochemical efficiency of PSII (Fv/Fm) of different lines under drought stress; (**E**) Water loss rate. Data are presented as means ± SD of three biological replicates. Significant differences were determined by Student’s *t*-test. Significance level: * *p* < 0.05, ** *p* < 0.01.

**Figure 4:**
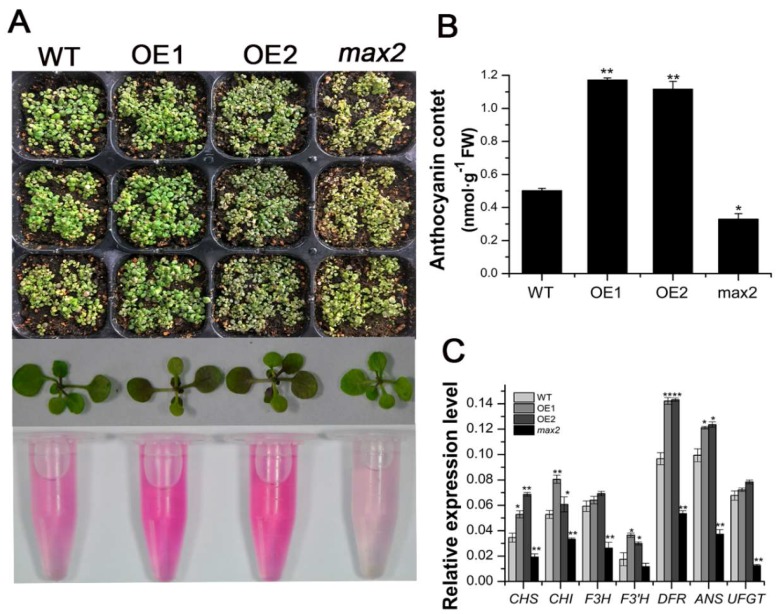
*SsMAX2* promoted anthocyanin accumulation in *Arabidopsis* leaves under drought stress. (**A**) Drought-induced anthocyanin accumulation in the leaves; (**B**) Anthocyanin level of different lines; (**C**) Relative expression of anthocyanin biosynthesis genes in the leaves of different lines in response to drought stress. Data are presented as means ± SD of three biological replicates. Significant differences between WT and the other groups were determined by Student’s *t*-test. Significance level: * *p* < 0.05, ** *p* < 0.01.

As the expression of *SsMAX2* in *S. sebiferum* seedlings was significantly induced by osmotic stress (Figure 2), we further investigated whether the constitutive expression of *SsMAX2* in *Arabidopsis* could confer drought and osmotic stress resistance. Results from the petri experiment showed that the *SsMAX2* OE lines exhibited significantly higher adaptation to osmotic stress, which was mimicked by the mannitol treatment (Figure 3A). Moreover, after withholding water for 11 days, all *Arabidopsis* lines exhibited significant dehydration, especially the wild-type (WT) and *max2* mutant seedlings. Seven days after re-watering, almost half of the *SsMAX2* OE lines survived (Figure 3B,C). Furthermore, chlorophyll fluorescence parameters, such as maximum photochemical efficiency of PSII (Fv/Fm), were investigated. The results showed that the *SsMAX2* OE lines exhibited much higher ratio of Fv/Fm under drought stress compared with the WT and *max2* mutant seedlings. Additionally, the water loss ratio in the leaf, which is an important characteristic of drought adaptation in plants, was much lower in the *SsMAX2* OE lines. These results suggest that *SsMAX2* positively regulates drought and osmotic stress adaptation in *Arabidopsis*. Interestingly, significant anthocyanin increase in the leaves of the *SsMAX2* OE lines was detected after drought treatment compared with the WT and *max2* mutant seedlings (Figure 4A). As previously reported, anthocyanin plays a key role in the regulation of the endogenous reactive oxygen species (ROS) level in response to abiotic stresses [21]. In this study, the leaves of two *SsMAX2* OE lines exhibited triple anthocyanin content compared to the WT and *max2* mutant seedlings (Figure 4B). Accordingly, the expression of the anthocyanin biosynthesis genes *chalcone synthase* (*CHS*), *chalcone isomerase* (*CHI*), *flavanone 3-hydroxylase* (*F3H*), *flavanone 3′-hydroxylase (F3′H*), *dihydroflavonol reductase* (*DFR*), and *anthocyanin synthase* (*ANS*) was more significantly upregulated in the *SsMAX2* OE lines and downregulated in the *max2* mutant in response to drought stress (Figure 4C), suggesting SL may also regulate anthocyanin biosynthesis. These results demonstrate that overexpression (OE) of *SsMAX2* confer drought and osmotic stress tolerance, and the significant upregulation of anthocyanin accumulation in the *SsMAX2* OE lines may contribute to drought and osmotic stress resistance in the *SsMAX2* OE lines.

### 2.3. SsMAX2 Conferred Salt Tolerance in Arabidopsis

**Figure 5:**
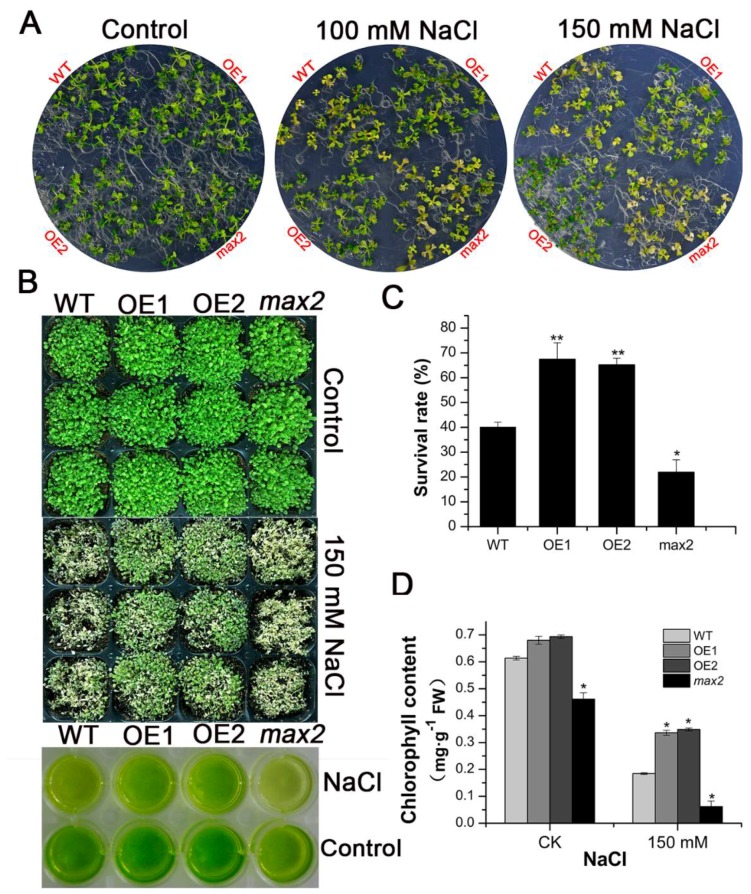
*SsMAX2* conferred salt resistance in *Arabidopsis*. (**A**) Growth phenotype of 5-day-old *SsMAX2* OE lines, *max2*, and WT seedlings under salt stress; (**B**,**C**) The growth and survival rate 7 days after 150 mM NaCl treatment on 15-day-old seedlings; (**D**) Chlorophyll content of the seedling leaves before or 7 days after salt stress treatment. Data are presented as means ± SD of three biological replicates. Significant differences between WT and the other groups were determined by Student’s *t*-test. Significance level: * *p* < 0.05, ** *p* < 0.01.

We further investigated the salt responses of different *Arabidopsis* lines (two *SsMAX2* OE lines, wild-type, and *max2* mutant). The results showed that seedlings of different lines exhibited no significant growth variations under normal conditions (Figure 5A). However, the WT and *max2* plants showed significant blushing phenotype after seven days of growth in half MS medium containing 100 mM NaCl, while the *SsMAX2* OE lines exhibited significantly higher tolerance to salt stress, even in the 150 mM NaCl medium (Figure 5A). The salt stress experiment was also conducted on different *Arabidopsis* lines growing in the soil. The results were in accord with that of the petri experiment, with the *SsMAX2* OE lines exhibiting robust salt tolerance (Figure 5B). The survival rate of the *SsMAX2* OE lines could reach as high as 65% at 7 d after 150 mM treatment (Figure 5C). It is worth noting here that the chlorophyll content of the *max2* mutant was significantly lower than that of *SsMAX2* OE and WT plants (Figure 5D). Stress can induce senescence and cause significant chlorophyll degradation in the leaf [22]. The results showed that the decrease in stress-induced chlorophyll in the leaves of the *SsMAX2* OE lines was significantly lower than that of the *max2* mutant and wild-type plants (Figure 5D). This suggests that *MAX2* may positively regulate chlorophyll synthesis and that *SsMAX2* overexpression in *Arabidopsis* can retard leaf senescence induced by salt stress.

### 2.4. SsMAX2 Promoted Seed Germination under Both Salt and Osmotic Stresses

**Figure 6:**
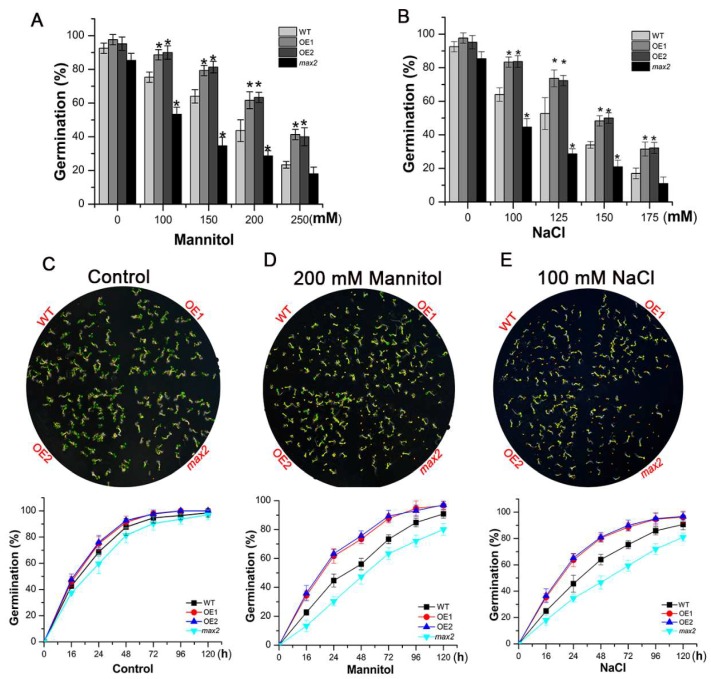
*SsMAX2* OE lines exhibited higher salt and osmotic stress tolerance during the seed germination stage. (**A**,**B**) Seed germination rate of the *SsMAX2* OE lines, *max2*, and WT under various concentrations of NaCl and mannitol treatment; (**C**–**E**) Time-course assay of the seed germination of different lines under 200 mM mannitol and 100 mM NaCl treatment. Data are presented as means ± SD of three biological replicates. Significant differences between WT and the other groups were determined by Student’s *t*-test. Significance level: * *p* < 0.05.

We further investigated whether the *SsMAX2* OE lines could also improve stress resistance during the seed germination stage. We evaluated the effects of different concentrations of mannitol and NaCl on the seed germination of different *Arabidopsis* lines. The results showed that, under normal conditions, the germination rate of *SsMAX2* OE1 lines, *max2*, and WT showed no significant variations (Figure 6A,B). However, the seed germination of WT and *max2* mutant was more likely to be inhibited, whereas *SsMAX2* OE lines exhibited much higher germination ratio, with increasing concentrations of both mannitol and NaCl (Figure 6A,B). The results also showed that, even under 200 mM mannitol and 150 mM NaCl, the seed germination of *SsMAX2* OE lines was still over 50% (Figure 6A,B). Furthermore, the time-course assay of the seed germination showed that the seed germination of the *max2* mutant was significantly delayed compared with that of the *SsMAX2* OE lines under both salt and drought stress (Figure 6C–E). These results suggest that *SsMAX2* confer significant salt and osmotic stress resistance during seed germination.

### 2.5. SsMAX2 Regulated the Hydrogen Peroxide, Malondialdehyde (MDA), Proline, and Soluble Sugar Accumulation in the Seedlings in Response to the Stresses

**Figure 7:**
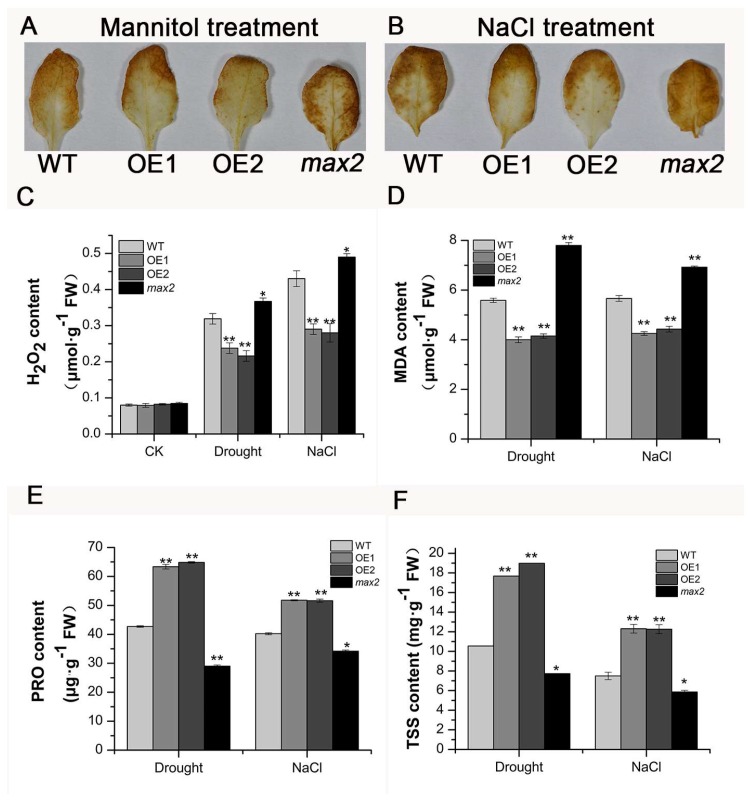
*SsMAX2* OE lines exhibited lower hydrogen peroxide and malondialdehyde (MDA) levels and increased proline and soluble sugar levels in response to osmotic and salt stresses. (**A**,**B**) DAB staining of the leaves of *SsMAX2* OE lines, *max2*, and WT after mannitol and NaCl treatment. The (**C**) hydrogen peroxide, (**D**) MDA, (**E**) proline, and (**F**) soluble sugar level of 15-day-old seedlings of the *SsMAX2* OE lines, *max2*, and WT were determined 5 days after withholding water or 150 mM NaCl treatment. Data are presented as means ± SD of three biological replicates. Significant differences between WT and the other groups were determined by Student’s *t*-test. Significance level: * *p* < 0.05, ** *p* < 0.01.

The significant increase in endogenous peroxide or superoxide chemical levels induced by the abiotic stresses is responsible for the initiation of leaf senescence and death [23]. Malondialdehyde (MDA) is an important marker for lipid peroxidation due to overproduction of ROS in the cell [24]. Here, the results showed that both osmotic and salt stress could cause a significant increase in hydrogen peroxide and MDA in all lines, while *SsMAX2* OE lines had a significant lower level of both hydrogen peroxide and MDA (Figure 7A–D), suggesting a tightly regulated ROS and MDA homeostasis in the *SsMAX2* OE lines. This was also in accord with the physiological results, which showed that *SsMAX2* OE lines had better resistance and delayed leaf senescence to the stresses.

Proline and soluble sugars play an important role in maintaining osmotic homeostasis in plant cells [23]. Our results showed that the *SsMAX2* OE lines accumulated higher proline and soluble sugars in leaves than WT and *max2* mutant (Figure 7E,F). As both drought and salt stress can break the osmosis homeostasis in the plant, the enhanced accumulation of proline and soluble sugars can significantly prevent water loss from leaves under osmotic stresses. The results show that decreased water loss in the *SsMAX2* OE lines contribute to drought and salt stress resistance.

### 2.6. SsMAX2 Increased the Enzyme Activity of Superoxide Dismutase (SOD), Peroxidase (POD), and Ascorbate Peroxidase (APX)

**Figure 8:**
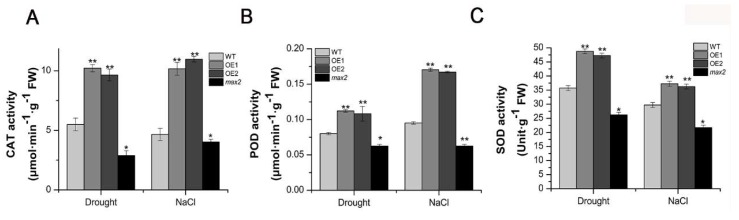
*SsMAX2* increased the activity of the antioxidant enzymes. The enzyme activities of (**A**) CAT, (**B**) POD, and (**C**) SOD of the extracts of 15-day-old seedlings were separately determined 5 days after withholding water or 150 mM NaCl treatment. Data are presented as means ± SD of three biological replicates. Significant differences between WT and the other groups were determined by Student’s *t*-test. Significance level: * *p* < 0.05, ** *p* < 0.01.

As the hydrogen level in the *SsMAX2* OE lines was significantly lower than that in WT and *max2* mutant, we further investigated whether the key enzymes involved in the regulation of ROS degradation were also affected in response to drought and salt treatment. POD, SOD, and CAT are the main oxidative enzymes involved in the regulation of ROS homeostasis in the cell [25,26]. The results showed that, under both drought and salt stress, the activity of POD, SOD, and CAT of the *SsMAX2* OE lines was significantly higher than that of *max2* and WT (Figure 8), whereas the max2 mutant exhibited the lowest activity of the antioxidative enzymes, further demonstrating a tightly controlled ROS scavenge ability controlled by SL signaling. These results suggest that *MAX2* may be involved in the regulation of plant ROS homeostasis via controlling the activities of oxidative enzymes.

### 2.7. Diverse Regulation of the Abscisic Acid (ABA) Biosynthesis Genes in SsMAX2 OE Lines and max2 in Response to Drought and Salt Stress

**Figure 9:**
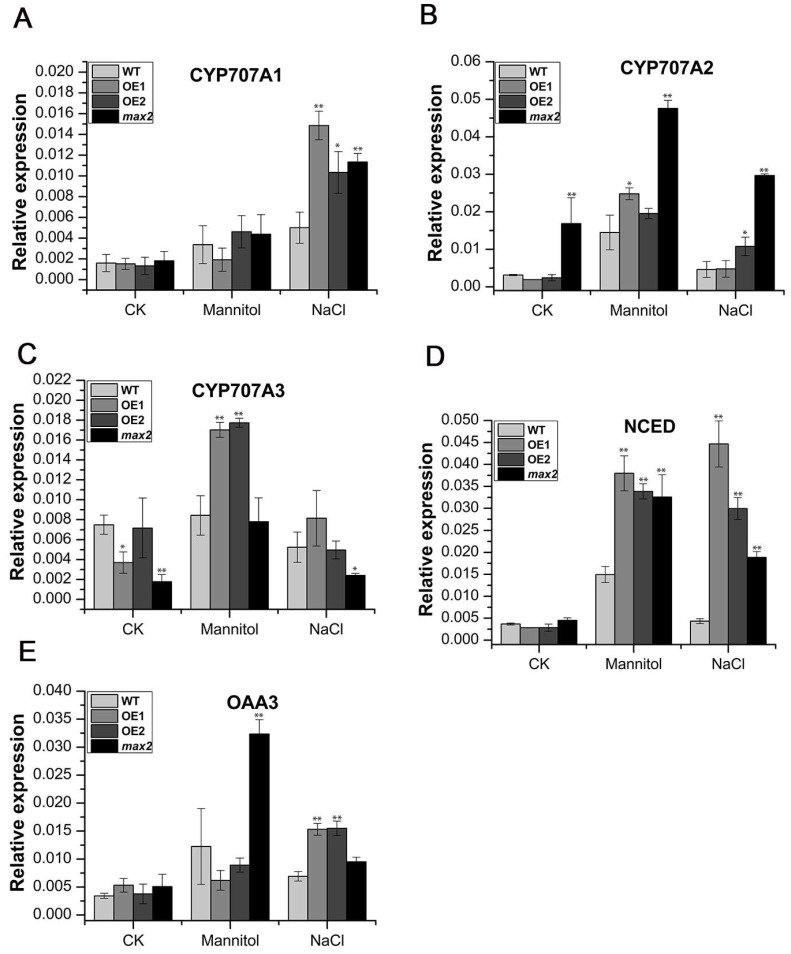
Expression of key ABA biosynthesis genes of the *SsMAX2* OE lines, *max2*, and WT was diversely regulated in response to salt and osmotic treatment. The gene expression of (**A**) *CYP707A1*, (**B**) *CYP707A2*, (**C**) *CYP707A3*, (**D**) *NCED3*, and (**E**) *OAA3* was determined at 6 h after mannitol and NaCl treatment by qPCR. *SsACT* was used as the internal control. Data are presented as means ± SD of three biological replicates. Student’s *t*-test was used to determine the significant differences between WT and other *Arabidopsis* lines. Significance level: * *p* < 0.05, ** *p* < 0.01.

ABA is the key phytohormone that directly regulates abiotic stresses. Thus, in this study, we further investigated whether the expression of ABA biosynthesis genes (*CYP707-A1*, *-A2*, *-A3*, *NCED3*, and *OAA3*) was diversely regulated in response to drought and salt stress. After salt treatment, the significant upregulation of *NCED3*, *OAA3*, and *CYO707A1* in the *SsMAX2* OE lines could be detected at 6 h after treatment compared with the WT and *max2* mutant (Figure 9). The expression of *CYP707A3* and *NCED3* was relatively higher in the *SsMAX2* OE lines (Figure 9C,D). Specifically, the basic expression of *CYP707A2* in the *max2* mutant was higher than the WT and *SsMAX2* OE lines (Figure 9B), which is in accord with previously published results in *Arabidopsis* [27]. These results suggest a potential interaction between SL and ABA in the regulation of abiotic stress adaptation.

## 3. Discussion

*Sapium sebiferum*, which is one of the most important commercial woody plants in China, has received considerable attention due to its high oil content in the seed coat and kernel, excellent sightseeing value as a landscape plant, and its high adaptation to the adverse marginal land. Recent studies on the plant have mainly focused on flower sex determination, seed yield, oil extraction and production, and herb values [28,29,30,31,32]. However, only few reports have demonstrated its antistress abilities. Abiotic stresses, such as drought and salt, can significantly reduce the yield output in *S. sebiferum* [33], which significantly limits its industrial potential. As the transgenic approaches have been widely used and proven to be very effective in the regulation of abiotic stress tolerance in many species [34,35,36], the generation of high-stress-resistant cultivars via genetic modifications is the foremost mission in *S. sebiferum* breeding.

Previous studies have demonstrated that SLs are involved in the regulation of shoot branching, senescence, and photomorphogenesis [37,38]. Some recent researches have revealed the pivotal role of SLs in the regulation of stress adaptation [39]. Thus, the identification and characterization of SL biosynthesis and signaling genes in woody plants are important for generating stress-tolerant cultivators. *More Axillary Branches 2* (*MAX2*), which encodes a F-box E3 ligase in *Arabidopsis*, is the key component involved in SL signal transduction [40]. *MAX2* plays a key role in the regulation of shoot branching, photomorphogenesis, and stress adaptation [13,27,41,42]. In this work, we identified and functionally characterized the *MAX2* homolog (*SsMAX2*) from the oil plant *S. sebiferum*. Constitutive expression of *SsMAX2* in *Arabidopsis* inhibited the shoot branching and the hypocotyl elongation (Figure 1), further confirming the biological functions of *SsMAX2* as *MAX2* homolog in *Arabidopsis*. In *S. sebiferum* seedlings, it was interesting to find that the expression of *MAX2* was significantly upregulated in response to drought and salt treatment (Figure 2). Many abiotic stress-inducible genes, such as *NAC5* [43], *XTH3* [44], and *UGT87A2* [45], are important in controlling the adaptation to stresses. Thus, the significant upregulation of *SsMAX2* indicates that it may be correlated with stress adaptation. In comparison with the *max2 Arabidopsis* mutant, our results further demonstrated that *SsMAX2* OE lines exhibited significant drought and salt tolerance (Figure 3 and Figure 5). The seed germination in the *SsMAX2* OE lines also had higher drought and salt tolerance (Figure 6), whereas the *max2* mutant was more sensitive to the stresses, as previously described [14,42]. These results demonstrate that *MAX2* participates in the regulation of stress adaptation. However, the regulatory mechanism of how *MAX2* controls the increased tolerance to abiotic stresses remains elusive.

Plants have developed many mechanisms to cope with biotic and abiotic stresses, such as accumulation of secondary metabolites, activated oxidative enzyme, or nonenzyme systems [36,46,47]. Anthocyanins, which consist of a group of phenolic compounds, act as important antioxidants in plants suffering from abiotic stresses [48]. Increased anthocyanin production has been shown to significantly enhance tolerance to abiotic stresses in *Arabidopsis*, grapevine, and bamboo [49,50,51]. Our results also showed that, under drought stress, significant accumulation of anthocyanins in the leaves of the *SsMAX2* OE lines was detected, while its content was relatively lower in the *max2* mutant and WT plants (Figure 4). The qPCR results further showed that the expression of the key genes in the anthocyanin biosynthesis pathway was significantly induced in the *SsMAX2* OE lines (Figure 4C). These results suggest that SLs may be involved in the regulation of anthocyanin biosynthesis in *Arabidopsis*, which is important for adaptation to salt stress. It is worth noting here that the *SsMAX2* OE lines had higher chlorophyll content in the leaves, while the *max2* mutant exhibited much lower chlorophyll content than WT plants under normal growth conditions (Figure 5). Furthermore, drought-stress-induced chlorophyll degradation in the leaves was much lower than that in WT and *max2* plants (Figure 5D), suggesting *MAX2* may positively regulate chlorophyll biosynthesis or accumulation, the level of which can be an important indicator of adaptation to abiotic stresses [52]. Many researchers have suggested that the accumulation of soluble sugars and amino acids is key for osmotic stress resistance [53,54] due to their direct role in the regulation of water uptake and loss. Our results also showed that the level of both proline and soluble sugars was significantly higher in the *SsMAX2* OE lines after drought and salt treatment in comparison with the WT and *max2* seedlings (Figure 7E,F), suggesting a pivotal role of *MAX2* in the regulation of cellular metabolite homeostasis.

The generation of oxidative chemicals induced by abiotic stresses in cells is the main cause of cell apoptosis and death [46]. Plants have developed a tightly regulated mechanism to maintain endogenous oxidative chemicals at a certain level. Many reports have demonstrated that exogenous application of ROS cleavage chemicals (e.g., melatonin) or overexpression of oxidative chemical cleavage enzymes (e.g., SOD, POD, and APX), can significantly promote tolerance to abiotic stresses due to the efficient cleavage of the stress-induced oxidative chemical level [55,56]. In this study, we presumed that *MAX2* could be involved in the regulation of oxidative chemical levels in plants. The results showed that both drought and salt treatment significantly induced hydrogen peroxide accumulation in *Arabidopsis* seedlings. However, the level of this was significantly lower in the *SsMAX2* OE lines compared with that of the *max2* mutant and wild-type (Figure 7A,B). Accordingly, the activity analysis of key oxidative enzymes, such as CAT, POD, and SOD, also proved that *SsMAX2* OE plants had higher capability in the cleavage of hydrogen peroxide induced by salt and drought stress, whereas *max2* mutant exhibited much lower enzyme activity (Figure 8). These results suggest that the SL signaling may be directly involved in the regulation of redox homeostasis, although the molecular mechanism still needs further investigation.

ABA is the key phytohormone that positively regulates abiotic stress adaptation. Many reports have demonstrated that ABA accumulation can happen immediately when plants are subjected to drought, salt, cadmium, or cold stresses [57]. Exogenous application with ABA or overexpression of ABA biosynthesis genes can significantly promote abiotic stress resistance in many species [58]. A recent study also demonstrated that ABA and SL coordinately regulated salt stress tolerance in *Sesbania cannabina* [59]. In this study, the *SsMAX2* OE lines exhibited higher expression level of key ABA biosynthesis-related genes, such as *CYP707A1*, *CYP707A3*, *NCED*, and *OAA3*, compared with the *max2* mutant or WT plants (Figure 9), further indicating that *SsMAX2*-induced stress tolerance may be partially ABA-dependent.

**Figure 10:**
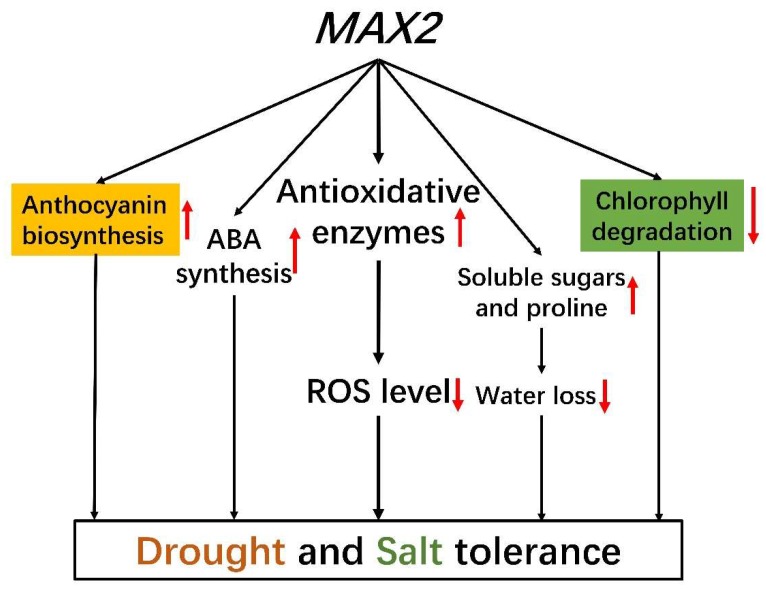
Model of *SsMAX2* in the regulation of drought and salt adaptation. The arrows indicate the downregulated or upregulated activities.

In this study, we isolated and functionally characterized the *MAX2* homolog in the oil plant *Sapium sebiferum*. We not only investigated the gene function in controlling shoot branching and hypocotyl elongation but, most importantly, we characterized the novel function of *SsMAX2* in the regulation of drought and salt adaptation. We showed that *MAX2* potentially controls chlorophyll biosynthesis and degradation, anthocyanin biosynthesis, soluble sugars, and proline accumulation (Figure 10). The physiological and biochemical results demonstrate that *SsMAX2* plays a pivotal role in the regulation of redox homeostasis via the regulation of antioxidative enzymes (Figure 10). The results also suggest that there may be potential interactions between SL and ABA in the regulation of abiotic stress adaptation (Figure 10). Further research will be focused on the identification of the molecular network of SL in the regulation of stress adaptation.

## 4. Materials and Methods

### 4.1. Plant Materials and Growth Conditions

The Col-0 ecotype *Arabidopsis* and *max2* mutant (CS9565) were ordered from the Arabidopsis Biological Resource Center (ABRC). *Arabidopsis* seeds were sterilized with 75% ethanol solution for one minute, followed by sterilization with 8% sodium hypochlorite for 15 min, and then germinated in half MS medium. *Arabidopsis* seedlings were grown in the growth chamber (22 °C; 16-h light/8-h dark photoperiod; 120 mol·m^−2^·s^−1^ radiation strength; 75% humidity). All plants were fertilized with half Hoagland solution every other week.

*Sapium sebiferum* seeds were germinated in the peat soil as previously described [60]. Two-week-old *S. sebiferum* seedlings were transplanted into the garden pot (10 cm × 10 cm) and grown in the chamber as described above.

### 4.2. Gene Cloning, Vector Construction, and Arabidopsis Transformation

The protein sequence of AtMAX2 (AT2G42620.1) was used to blast against the local *S. sebiferum* transcriptome database using the NCBI local blast package: BLAST 2.7.1. The coding sequence (CDS) and amino acid sequence information is listed in Table S2. The complete CDS of *SsMAX2* was cloned into the pOCA30 expression vector and transformed into the *Agrobacterium* EHA105. The *Arabidopsis* transformation was carried out following a previously described method [61]. The candidate transgenic *Arabidopsis* plants were firstly screened on half MS containing 40 mg/L kanamycin and then further confirmed by RT-PCR. Two out of over 10 independent homozygous transgenic lines were used for further experiments.

### 4.3. Drought and Salt Treatment

In the petri experiment, different concentrations of mannitol and NaCl were separately used for drought and salt stress treatment. In the soil experiment, plants of different lines were grown in moss peat soil. To induce drought conditions, water was withheld for a number of days, as indicated in the figures. The plant growth was monitored after three days after rewatering. For salt stress, the pods of *Arabidopsis* were directly submerged in different concentrations of sodium chloride until the soil was completely saturated. Then, the plants were put under normal growth conditions.

### 4.4. RNA Extraction and Quantitative Real-Time PCR (qPCR)

Total RNA was extracted from *Arabidopsis* and *S. sebiferum* seedlings according to the instructions of HP Total Plant RNA kit (Omega, Shanghai, China). RNA concentration and integrity were further analyzed by a micro-analyzer and gel electrophoresis. For cDNA synthesis, 1–1.5 g of total RNA was used using the TransScript II One-Step gDNA removal and cDNA Synthesis SuperMix (Transgen, Beijing, China). qPCR was performed using the Premix Ex Taq^TM^ II (Transgen, Beijing, China) on the LightCycler 96 System (Roche, Basel, Switzerland). The qPCR program was set as follows: preheating, 95 °C, 10 min; amplification (45 cycles), 95 °C, 10 s, 60 °C, 20 s, and 72 °C, 20 s; melting curve: 95 °C, 2 min, 60 °C, 30 s, then continuously increased to 95 °C. The calculation of the relative gene expression was based on the 2^−∆∆*C*q^ method as described previously [62]. The detailed primer information for each gene is listed in Table S3.

### 4.5. Total Chlorophyll and Anthocyanin Determination

The total chlorophyll content was analyzed based on a previously described method [63]. The leaf tissue was homogenized in liquid nitrogen and subsequently extracted in 80% acetone containing 1 M KOH overnight. After centrifugation at 12,000× *g* for 10 min, the supernatant was used for chlorophyll determination using a Scandrop spectrophotometer (Analytikjena, Jena, Germany). The anthocyanin was determined as previously described [64] with minor modifications. Briefly, the *Arabidopsis* leaves were ground into fine powders in liquid nitrogen. Then, the powders were transferred to methanol containing 1% HCl at 4 °C in the dark for 24 h. The aqueous phase was then used for anthocyanin determination in the spectrophotometer with the following formula: OD = (A_530_ − A_620_) – 0.1 (A_650_ − A_620_).

### 4.6. Determination of the Water Loss Rate

Approximately 0.5 g fresh leaves of 15-day-old *Arabidopsis* plants were collected and weighed immediately. The leaves were kept in a petri dish in open air. The water loss rate was calculated every hour based on the change in leaf weight, as previously described [65].

### 4.7. Diaminobenzidine (DAB) Staining of Hydrogen Peroxide in the Leaves

Diaminobenzidine (DAB) staining was used for in situ detection of hydrogen peroxide in *Arabidopsis* leaves as previously described [56]. The detached *Arabidopsis* leaves of different *Arabidopsis* lines were submerged in the DAB solution (1 mg·mL^−1^, pH 3.8) overnight at room temperature. The leaves were submerged in ethanol until the chlorophyll was washed off. Then, the leaves were used for hydrogen peroxide detection.

### 4.8. Determination of Hydrogen Peroxide, MDA, Proline and Total Soluble Sugar Level, and Antioxidant Enzyme Activity

The plant samples were firstly ground into powder in liquid nitrogen and then suspended in ice-cold phosphate buffer (0.1 M, pH = 7). The sample was vortexed at maximum speed for 1 min and centrifuged at 12,000 rpm for 15 min. The supernatants were then used for further determination of the hydrogen peroxide level and antioxidant enzyme activity. The hydrogen peroxide level of different samples was determined using the Hydrogen Peroxide Assay Kit (Jiancheng Bioengineering Institute, Nanjing, China). The absorbance at 405 nm was determined by a Scandrop spectrophotometer (Analytikjena, Germany). The hydrogen peroxide level was calculated based on a previously described formula [56]. The MDA, proline, and total soluble sugar level were separately determined using the MDA Assay Kit, the Proline Assay Kit, and the Plant Soluble Sugar Content Test Kit (Jiancheng Bioengineering Institute, Nanjing, China), as previously described [24].

The antioxidant enzyme activities were also determined using the spectrophotometric method. SOD, POD, and CAT activities were separately determined using the Total Superoxide Dismutase (T-SOD) Assay Kit, Peroxidase Assay Kit, and Catalase (CAT) Assay Kit (Jiancheng Bioengineering Institute, Nanjing, China), as previously described [56].

### 4.9. Chlorophyll Fluorescence Measurement

The photosynthesis rate of the *Arabidopsis* plants of different lines after drought treatment was analyzed using the Portable Photosynthesis Rate Detector AGHJ-PPF (Anhui Institute of Optics and Fine Mechanics, Chinese Academy of Sciences) after a 30 min dark adaptation, as previously described [66]. Parameters including the maximum photochemical efficiency of PSII (Fv/Fm), minimal fluorescence (F0), maximal fluorescence (Fm), and PSII (as shown in Figure S1) were calculated according to a previous method [66].

### 4.10. Phylogenetic Analysis

The homologs of MAX2 of the other species were obtained by blasting against nucleic acid sequences in GenBank. Phylogenetic analysis was carried out using MEGA software Version 7.0 [67]. One thousand bootstrap replicates were performed for the phylogenetic tree construction.

### 4.11. Statistical Analysis

Multiple comparisons between different samples were carried out using Statistical Product and Service Solutions (SPSS, Chicago, IL, USA) with one-way ANOVA, followed by the Tukey’s test (*p* < 0.05). Student’s *t*-test was used to analyze the significant difference between the indicated groups and control.

## Supplementary Materials

Supplementary materials can be found at http://www.mdpi.com/1422-0067/20/4/837/s1. Table S1: Information of the MAX2 homologs from other species. Table S2: CDS and amino acid sequence information of SsMAX2. Table S3: Information of all the primers used in this study. Figure S1: Parameters of the photosynthesis rate analysis by the Portable Photosynthesis Rate Detector AGHJ-PPF.

## Abbreviations

ABA	Abscisic acid
MDA	Malondialdehyde
MS	Murashige and Skoog
OE	Overexpression
PEG	polyethylene glycol
qPCR	Quantitative real-time PCR
ROS	Reactive oxygen species
rpm	Round per minute
RT-PCR	Reverse transcription PCR
SL	Strigolactone
WT	Wild-type
