# Supplementary material for: Overexpression of the Stress-Inducible SsMAX2 Promotes Drought and Salt Resistance via the Regulation of Redox Homeostasis in Arabidopsis
Source: Int J Mol Sci. 2019 Feb 15;20(4):837. doi: 10.3390/ijms20040837 (PMC6412474; doi:10.3390/ijms20040837)
Supplement: Supplementary file 1 [file ijms-20-00837-s001.zip › Table S2. CDS and amino acid sequence information of SsMAX2.docx]

Table S2. CDS and amino acid sequence information of SsMAX2

CDS sequence:

ATGGCCGCACCAACCGCCGCAACCACCATCCACGACCTCCCCGACGTTATTTTGTCTAATGTTATTGCTTCCATTTCAGATACTCGAACCAGAAACTCAATTTCTCTGGTTAGCCGGAAATTTCTCACTCTTGAAAGAGCTACTCGCACTAACCTCACTCTCCGTGGCAATTCAAGGGACCTTTACATGATTCCTACTTGTTTCAGATCTGTCACTCATCTTGATCTCTCTTTGCTCTCTCCATGGGGTCATTATTTTCTCTCTTCTTCTCACCCCTCTGATCCTCTCCTCCTCGCCCACCGCCTCGCTGTGGCGTTTCCTCTTGTTACTTCTCTCACCCTCTATTGCCGCTCTCCTTCCACTCTCCAGATCCTCCTTCCACGGTGGCCGATGTTGACTTGTATGAAGCTAATTCGGTGGCATCAGCGTCCGTCTTCGTCTCATCTCGGGGCTGATTTTATTCCTTTGCTCGAGCAATGTCAGTTGCTCACTTGTCTCGATCTCTCGAATTTCTATTACTGGACGGAGGATTTGCCTCCTGTTCTTCAAGCTTATCCTGATGTATCGAAATCATTGACTTGCTTGGATCTCCTCACTGTTTCGCTAACTGATGGATTTAAGGCAGATGAGATTCGAACAATCACTGCCGCTTGTTCAAATCTCAGGAAGTTTCTTGTTGCTTGTATGTTCGATCCGAGTTATCTTGGATTTGTAGGAGACGAGACGTTGCTTGCTATTGCTGCGAATTGTCCCAGGTTATCGGTTCTTCATCTGGTAGATACGGCGTCGTTGGGGAGCACGAGGAATGATCCTGAAGACGAAGGGTTAACTGGGGAGGATGCTAGGGTTACAGTTGCTGGATTAGTTGATTTTTTCTCTGGCCTTCCGTTGCTGGAAGAGTTGTTTTTTTGTGTTTGTAAAAGTGTTAGAGATACTTCTGTGGCTCTTGAAGCGCTTAATTCTCGGTGTCCGAAGCTCAAAGTGCTCAAGTTAGGGCAGTTCCACGGCATTTGTATGGCTGTTGAGTCGCAGCTCGATGGGGTTGCTTTGTGTTCGGGGCTGAGATCTTTGTCCATTACCAAGTCCGCGGATTTGACGGATATGGGTTTGATTGAGATTGGTAGAGGATGTTGTAAACTTGCGAAGTTTGAAGTTGAAGGTTGCAAGAAGATTACTATGAAGGGGATGAGGACAATGGCTTGTTTGCTTCACAAGACTCTGGTTGAGGTCAAGATCTCTGCTTGTAAGAATCTCAATGCTGTAGCGTCTTTGCAAGCTTTGGAGCCTATTCGTCACCAGATTGAGTCTCTTCATATCGATTGTATTTGGAATGCTGTGCAACAAGAGGAAAATTATGCTGCTTATCATTGTTTCGATCTCAATGAAGACCTCTTTGAAGGTGCTCAAGATGAGTATTGCAACAAGAGTAAGAGAATTAAGTATTCAGATGAAGCTTGTTTTATGCAAAGCAATGGGGATGGATTTTGGAGCAAGTCCTGGGATAAACTTAGAGTTCTCTCAATCTGGATTGCTGTAGGTGAGCTTCTAACTCCATTGCCAATGGCTGGCCTCCAGGATTGTCCTCGTTTGGAGGAGATTCGGATACAGGTTGAAGGGGATTGCAGGGGCCGGCATAAACCATCACAGCCTGCATTTGGATTGAGCTGCCTTGCACATTATCCTTGTTTGTCAAAGATGCAGTTGAATTGTAGTGACACAATAGGTTTTGCTTTAACTGCCCCCTCTGGACAAATGGATTTGAGCCTGTGGGAGAGGTTTTTCTTGAATGGAATAGGGAATTTGAATCTCAACGAGCTTGATTATTGGCCACCACAAGACCGGGATGTTAACCAGAGGAGTTTATCACTTCCAGGAGCTGGGTTGCTTGCACAATGTCTTTCACTAAGAAAGCTCTTTATACATGGAACAGCACATGAGCATTTCATGATGTTTCTTCTCCGAATTCCTAACCTGAGGGATGTGCAACTTAGGGAAGACTACTATCCCGCACCAGAGAATGATATGAGCACTGAGATGAGAGTGGGATCATGCAGTCGGTTCGAAGATGCCTTGAACGGGCGCCAAATTCTGGACTGA

Amino acid sequence:

MAAPTAATTIHDLPDVILSNVIASISDTRTRNSISLVSRKFLTLERATRTNLTLRGNSRDLYMIPTCFRSVTHLDLSLLSPWGHYFLSSSHPSDPLLLAHRLAVAFPLVTSLTLYCRSPSTLQILLPRWPMLTCMKLIRWHQRPSSSHLGADFIPLLEQCQLLTCLDLSNFYYWTEDLPPVLQAYPDVSKSLTCLDLLTVSLTDGFKADEIRTITAACSNLRKFLVACMFDPSYLGFVGDETLLAIAANCPRLSVLHLVDTASLGSTRNDPEDEGLTGEDARVTVAGLVDFFSGLPLLEELFFCVCKSVRDTSVALEALNSRCPKLKVLKLGQFHGICMAVESQLDGVALCSGLRSLSITKSADLTDMGLIEIGRGCCKLAKFEVEGCKKITMKGMRTMACLLHKTLVEVKISACKNLNAVASLQALEPIRHQIESLHIDCIWNAVQQEENYAAYHCFDLNEDLFEGAQDEYCNKSKRIKYSDEACFMQSNGDGFWSKSWDKLRVLSIWIAVGELLTPLPMAGLQDCPRLEEIRIQVEGDCRGRHKPSQPAFGLSCLAHYPCLSKMQLNCSDTIGFALTAPSGQMDLSLWERFFLNGIGNLNLNELDYWPPQDRDVNQRSLSLPGAGLLAQCLSLRKLFIHGTAHEHFMMFLLRIPNLRDVQLREDYYPAPENDMSTEMRVGSCSRFEDALNGRQILD
